# Curettage of benign bone tumors without grafts gives sufficient bone strength: A case-series of 78 patients

**DOI:** 10.1080/17453670902804604

**Published:** 2009-02-01

**Authors:** Takashi Yanagawa, Hideomi Watanabe, Tetsuya Shinozaki, Kenji Takagishi

**Affiliations:** ^1^Department of Orthopaedic Surgery, Gunma University Graduate School of MedicineMaebashiJapan; ^2^Department of Physical Therapy, Gunma University School of Health ScienceMaebashiJapan

## Abstract

**Background and purpose** The defect that results after curettage of a bone tumor is usually filled in the same way. We report the outcome in patients with benign bone tumors that were treated with curettage but no filling.

**Patients and methods** We retrospectively studied 78 patients (mean age at the time of operation was 27 (6–73) years, 44 men) who had had a benign bone tumor curetted with no filling of the defect. The commonest tumor types were giant cell tumor of bone (27), fibrous dysplasia (13), enchondroma (9), and simple bone cyst (7). The mean size of the lesions was 35 (2–196) cm^3^. Normal activities, including full weight bearing for lower extremity lesions, were allowed after 3 months or less. The patients were followed for an average of 10 (1.2–21) years.

**Results** A postoperative fracture with a minor displacement occurred in 3 patients, in 2 of them because of local recurrence. All fractures healed. Local recurrence occurred in 9 patients; 7 of them had a giant cell tumor. Repeated local recurrences necessitated above-knee amputation in 1 patient. All other patients had unrestricted activities of daily living.

**Interpretation** Routine filling of curetted bone lesions does not appear to be necessary from a mechanical point of view.

## Introduction

Benign bone tumors are often treated with intralesional curettage, which creates a bone defect. To fill the defect, autogenous bone (Bickels et al. 1999), polymethylmethacrylate cement (Campanacci et al. 1990, Bini et al. 1995, Wada et al. 2002, Kivioja et al 2008), hydroxyapatite (Matsumine et al. 2004), and tricalcium phosphate (Hirata et al. 2006) are often used. The reason for filling the defect seems to be to increase final bone strength and, especially for giant cell tumors, to reduce the risk of local recurrence.

Enchondroma in the hand or foot can be successfully treated without filling (Hasselgren et al. 1991, Goto et al. 2002, 2004), however. Chigira et al. (1992) curetted benign bone tumors in different locations without filling and, even in large defects, they found bone remodeling with sufficient strength for daily activity. Waldram and Sneath (1989) stated that bone grafting is not necessarily required after curettage of a giant cell tumor. Otherwise, few reports regarding this procedure have been published.

We retrospectively examined the outcome in patients with a benign bone tumor treated with curettage without filling.

## Patients and methods

### Patients

We retrospectively reviewed patients treated between 1987 and 2007. The inclusion criteria were histologically confirmed benign bone tumors (except for tumors in the hands or feet) treated with curettage without any type of filling, and a minimum of 1 year of follow-up. Patients who received adjuvant chemotherapy or radiotherapy were excluded (they had metastasizing giant cell tumors).

78 patients (44 males) met the criteria for inclusion. Their mean age at the time of the operation was 27 (6–73) years. The mean follow-up period was 10 (1.2–21) years. The sites of the lesions were femur (33), lower leg bones (25), forearm bones (7), pelvic bones (6), humerus (6), and clavicle (1). The diagnoses were giant cell tumors of bone (27), fibrous dysplasia (13), enchondroma (9), simple bone cyst (7), non-ossifying fibroma (6), chondroblastoma (5), chondromyxoid fibroma (4), xanthofibroma (3), desmoplastic fibroma (2), benign fibrous histiocytoma (1), and osteofibrous dysplasia (1). Of the 27 giant cell tumors, 6 were radiographic grade-I lesions, 10 were grade-II, and 11 were grade-III, according to Campanacci et al. (1987). The lesion in 16 of the 78 patients was located within 15 mm of the surface of a weight-bearinjoint: hip, knee, or ankle.

The volume of defects was calculated as: width/2 × depth/2 × height × π for defects of cylindrical form and width/2 × depth/2 × height/2 × 4/3π for spherical defects (Hirn et al. 2008). The mean volume of the tumors was 35 (2–196) cm3. 10 patients had a preoperative fracture (bone cyst (3), giant cell tumor (2), fibrous dysplasia (2), chondroblastoma (1), desmoplastic fibroma (1), osteofibrous dysplasia (1)). Their lesions had a mean size of 48 (2–196) cm^3^ (unpaired t-test, p = 0.2 compared to lesions without fracture).

### Operative technique and rehabilitation

A cortical fenestration sufficiently large to be able to visualize the whole tumor cavity was made with an osteotome. The tumor was removed with curettes followed by enlargement of the cavity with a high-speed burr. The cavity was irrigated with sterile saline and was not filled in. All patients were allowed to begin range-of-motion exercises immediately after the operation. Orthoses for extremities were used for not more than 3 months. Patients with a lesion on the femur or tibia used no weight or partial weight for not more than 3 months after the operation. Patients with a pelvic lesion were allowed partial weight bearing using a walking frame or crutches 3 weeks postoperatively. Plain radiographs were taken postoperatively and then every 3 months for 2 years. MRI or CT examinations were made at 6- or 12-month intervals to evaluate bone formation and detect local recurrence for 2 years.

## Results

**Figure 1.**
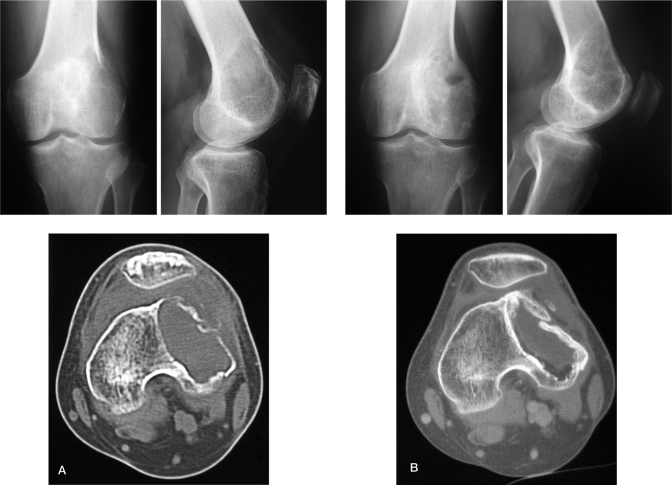
A 34-year-old male with a giant cell tumor in the left femur. A. Computed tomography showed a thin wall around the cavity 1 month after the curettage. B. 1 year postoperatively, bone formation was excellent and the cortex of the cavity was thicker than the normal cortex of the medial condyle.

**Figure 2.**
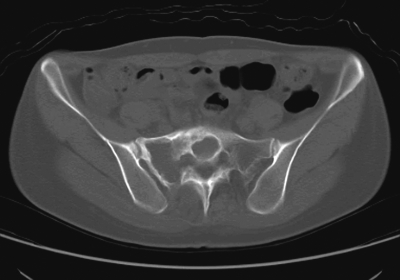
A 32-year-old female with fibrous dysplasia in the sacrum. More than half of the sacral body was occupied by the tumor, which invaded the sacral canal to a slight degree. Computed tomography showed good bone formation of the curetted wall 1 year after the operation.

None of the patients developed a postoperative infection. The lesions began to show bone formation on radiographs at 3 months: the thickness of the cavity wall increased and the cavity gradually became radiopaque, although complete bone filling of all cavities was not achieved (Figures 1 and 2).

A postoperative fracture with a minor displacement occurred in 3 patients: 1 giant cell tumor, 1 osteofibrous dysplasia, and 1 fibrous cortical defect. The giant cell tumor was a 31-cm^3^ grade-III lesion in the distal femur, which fractured at 10 months after the operation. A local recurrence was revealed by MR. Repeated local recurrences finally required an amputation. The osteofibrous dysplasia was a 13-cm^3^ lesion in the tibia. A fracture through a local recurrence occurred 12 months after the operation. The fracture healed with closed treatment, after which curettage was performed The diagnosis was fibrous dysplasia at the first operation but it was changed to osteofibrous dysplasia after the second, which means that the patient should probably not have been operated (Campanacci and Laus 1981). The fibrous cortical defect was a 3-cm^3^ lesion of the fibula and the operation was performed because of severe pain and strong anxiety of the patient concerning the disease. The patient was allowed to bear full weight just after the operation and had a fracture 3 weeks later, which healed without treatment.

Local recurrence occurred in 9 patients, 7 of whom had a giant cell tumor (altogether, 27 patients had a giant cell tumor); another had a simple bone cyst and one had an osteofibrous dysplasia. The mean time to recurrence was 22 (5–47) months. Local recurrence occurred in 1 of 6 grade-I giant cell tumors, in 1 of 10 grade-II, and in 5 of 11 grade-III tumors. Of the 9 patients with local recurrence, 1 patient with a giant cell tumor (of grade III) eventually required an amputation above the knee after repeated local recurrences. 1 patient with a giant cell tumor (grade III) of the proximal humerus had an en bloc resection and an intramedullary rod was secured into the remaining humerus and fastened proximally to a clavicle as a functional spacer (Kaelin and Emans 1985). 1 patient with a bone cyst was treated with injection of steroid into the cyst, and the other 6 patients underwent re-curettage without bone grafts. All of the patients were disease-free at the latest follow-up.

At the latest follow-up, the patient who had been amputated above the knee after repeated recurrences of giant cell tumor needed a prosthesis. All other patients had unrestricted activities of daily living. There was only 1 case with degenerative changes in the joint adjacent to the lesion. This was a giant cell tumor of the tibia with 4 local recurrences.

## Discussion

Many authors recommend that after curettage of benign bone tumors the defect should be filled with bone grafts or substitutes such as cement, hydroxyapatite, or tricalcium phosphate. However, the necessity and risk of using such materials are poorly documented. Autogenous bone grafting has the advantages of there being no immunological reaction and good bone induction, but sufficient bone for large defects may be difficult to obtain and may give sequelae at the donor site. Packing of defects with methylmethacrylate cement gives immediate stability, especially for lesions close to a joint (Frassica et al. 1990). The thermal effects of cement polymerization induce some millimeters of bone necrosis, which may kill residual tumor cells (Mjöberg et al. 1984)—but this has also been suspected of damaging cartilage and subchondral bone in lesions close to a joint, which may be followed by degenerative arthritis (Campanacci et al. 1990, Bini et al. 1995). However, a recent long-term follow-up study of cemented giant cell tumors did not show that there was a high risk of osteoarthritis development (Vult von Steyern et al. 2007). Hydroxyapatite has been used to fill cavities after curettage (Itokazu et al. 1996, Matsumine et al. 2004) but it may interact with human monocytes, leading to the release of inflammatory cytokines (Laquerriere et al. 2003, Grandjean-Laquerriere et al. 2007). Successful filling of bone defects with β-tricalcium phosphate has been reported by Hirata et al. (2006). However, tibial cyst formation has been reported in an anterior cruciate ligament reconstruction using a bioabsorbable screw made of β-tricalcium phosphate and poly L-lactide (Malhan et al. 2002). Grafting with hydroxyapatite (Hamson et al. 1995, Matsumine et al. 2004) or β-tricalcium phosphate (Hirata et al. 2006) requires several months for bone incorporation. Only cement filling gives a degree of mechanical strength that is sufficient for immediate weight bearing. The lack of difference in required convalescence time between grafting with biodegradable materials and leaving the cavity empty compelled us to question the practice of filling the cavity with substitutes that may evoke foreign body reactions.

We found that curettage without filling of the defect led to a degree of bone strength that was sufficient for the activities of daily living in the majority of patients. We did not try to measure the amount of bone formation and remodeling; the exact radiographic appearance is of minor interest, as long as the strength of the bone permits normal activities. A postoperative fracture with minor displacement occurred in 3 cases; 1 of them could probably have been prevented with limitation of postoperative weight bearing, and the 2 others occurred in local recurrences.

In our series, local recurrence occurred in only 2 of the 51 tumors that were not giant cell tumors. 9 local recurrences occurred in the 27 giant cell tumors, however, 5 of them in 11 radiographic grade-III tumors. To prevent local recurrence of giant cell tumors, adjuvant therapies may be used after curettage. Filling of the cavity with bone cement gives immediate mechanical strength and is also thought to kill residual tumor cells by the heat generated during the polymerization, thereby reducing the local recurrence rate. However, the recurrence rate after cementation ranges from excellent to equivalent to that after curettage without cement (O'Donnell et al. 1994, Turcotte et al. 2002, Vult von Steyern et al. 2006, Kivioja et al. 2008). The necrotic effect of cement on the local recurrence rate seems to be unclear. Liquid nitrogen has been used as an adjuvant treatment for giant cell tumors after curettage because of its ability to induce osteonecrosis to a depth of 7–12 mm (Malawer et al. 1988). This treatment has been reported to reduce the local recurrence rate; however, the rate of postoperative fractures is high due to difficulty in controlling the amount of necrosis induced (Jacobs and Clemency 1985, Bickels et al. 1999, Malawer et al. 1999). Phenol has also been used to kill residual tumor cells after curettage of giant cell tumors, but the recurrence rates have been reported to show no improvement (McDonald et al. 1986, Turcotte et al. 2002). Extensive curettage of the tumor may be more important than the use of adjuvant therapies (Blackley et al. 1999, Eckardt and Grogan 1986).

In summary, we consider curettage without filling of the defect to be a standard treatment for benign bone tumors.
